# Supplementary material for: Pandemic Vibrio cholerae shuts down site-specific recombination to retain an interbacterial defence mechanism
Source: Nat Commun. 2020 Dec 7;11:6246. doi: 10.1038/s41467-020-20012-7 (PMC7721734; doi:10.1038/s41467-020-20012-7)
Supplement: Supplementary file 1 — Supplementary Information [file 41467_2020_20012_MOESM1_ESM.pdf]

Supplementary Information

Supplementary Figures and Tables

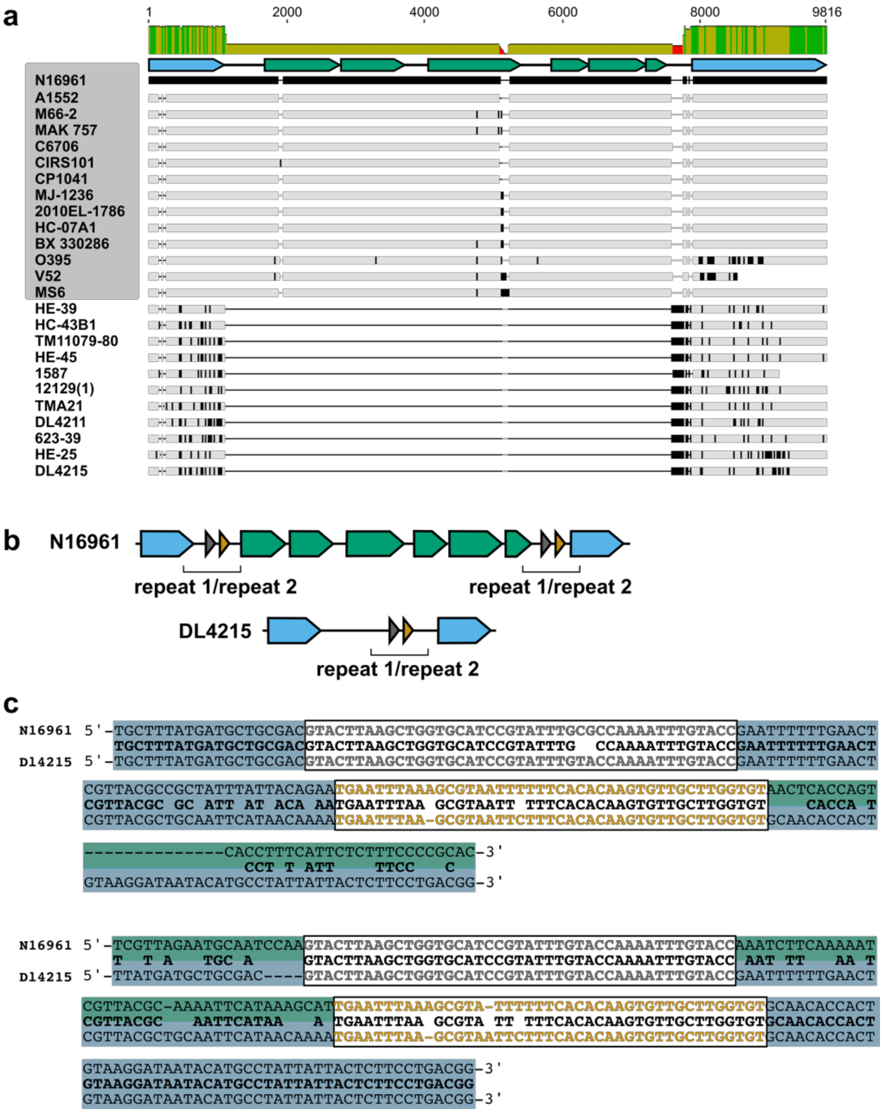

Supplementary Fig. 1: Aux3 was likely integrated site-specifically at repeat 2 in the *V. cholerae* chromosome. **a** MAUVE alignment of the Aux3 locus in pandemic (grey background) and environmental (white background) *V. cholerae* strains. N16961 is set as the reference sequence. Black indicates disagreement to the reference. Identity is represented in bars above the alignment (green = 100%, yellow = 99%-30%, red = <30%). Aux3 schematic is shown under the identity graph (blue = genomic flanks, green = Aux3 genes). **b** Schematic of Aux3 *att* sites in both the integrated (N16961) and naïve (DL4215) state. Genomic flanking genes are shown in blue. Aux3 genes are shown in green. Repeat 1 is shown in grey and repeat 2 is shown in orange. **c** MUSCLE alignment of the upstream (top) and downstream (bottom) Aux3 flanking repeats in N16961 to the naïve repeats in DL4215 (top/bottom). Matching bases are shown in bold black. Repeats are boxed and

bolded (repeat 1 in grey, repeat 2 in orange). Genomic flanks are highlighted in blue. Aux3 module is highlighted in green.

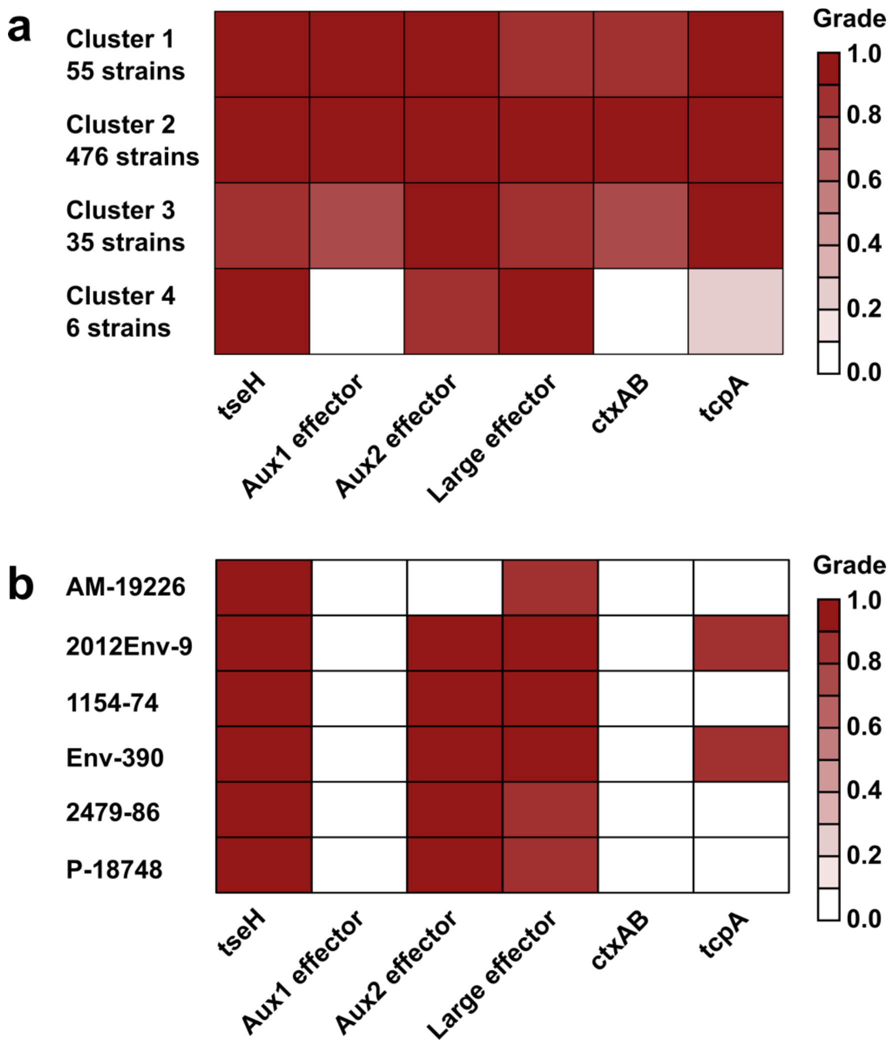

Supplementary Fig. 2: The *tseH* gene is found in pandemic *V. cholerae* and a small environmental reservoir. **a** Heatmap of 572 *tseH* (+) *V. cholerae* genomes from the PATRIC database. Genomes are collapsed into clusters based on mean BLAST Grade for *tseH* and five other pandemic associated factors: *tseL* (Aux1), *vasX* (Aux2), *vgrG3* (Large), *ctxAB* (CT), and *tcpA* (TCP). **b** Expanded heatmap of the six strains assigned to cluster 4. Lack of *tseL*, *ctxAB*, and *tcpA* indicates that these are non-pathogenic environmental strains.

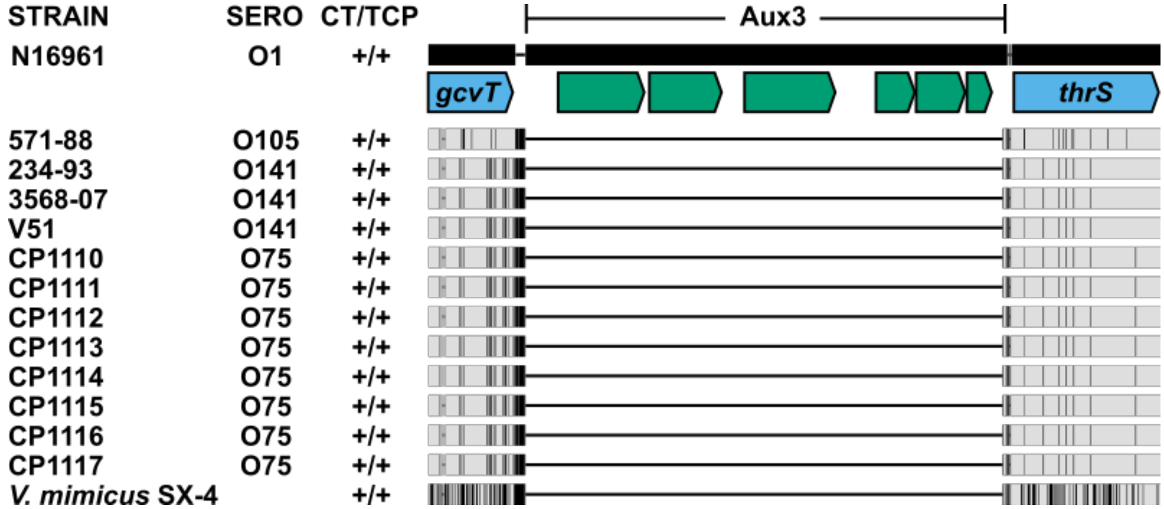

Supplementary Fig. 3: Aux3 is absent from non-pandemic, pathogenic *V. cholerae* and *V. mimicus* strains. MAUVE alignment of the Aux3 locus (VCA0280-VCA0287) in N16961 and 13 nonO1/O139, CTX+/TCP+ *V. cholerae* and *V. mimicus* strains that have been shown to cause isolated disease. N16961 is set as a reference sequence and differences against the reference are shown below. Grey bars indicate agreements and black bars indicate differences.

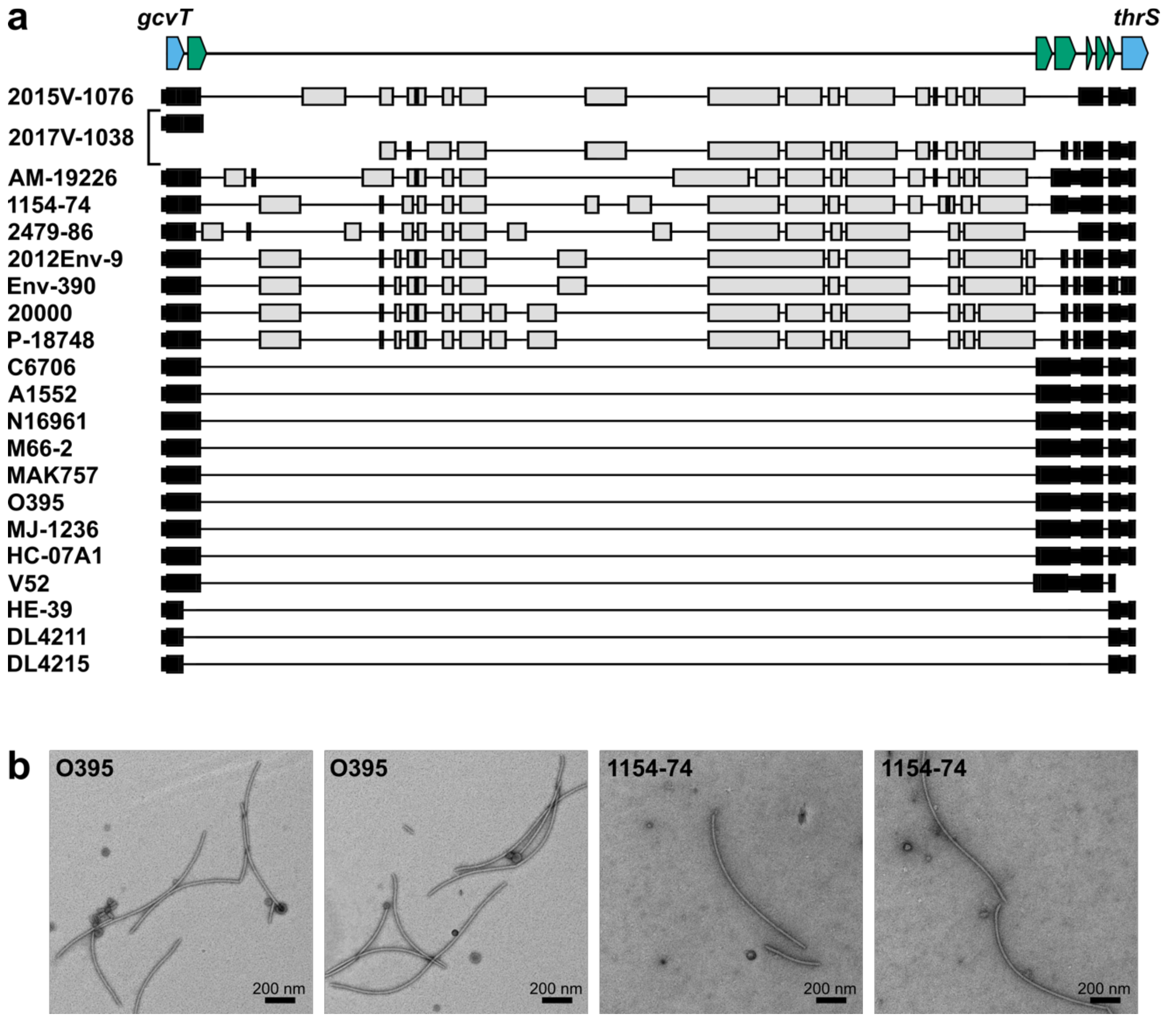

Supplementary Fig. 4: Environmental Aux3 strains encode variable extra sequence between *int* and VCA0283. **a** MAUVE alignment of the Aux3 locus (VCA0280-VCA0287) for 9 Aux3-encoding environmental strains, 9 Aux3-encoding pandemic strains, and 3 Aux3-naïve strains. Black bars indicate nucleotides conserved in the pandemic Aux3-encoding strains. Grey bars indicate nucleotides absent from the pandemic Aux3 element. Pandemic Aux3 schematic is shown (top) with genomic flanking genes in blue and Aux3 genes shown in green. **b** Electron micrographs of bacteriophage preparations from the supernatants of *V. cholerae* O395 (positive control preparation of CTX phage) and *V. cholerae* 1154-74. Scale bars are indicated. Electron micrographs for each sample are two representatives of ten images from each phage preparation. Phage preparations were generated once (n=1).

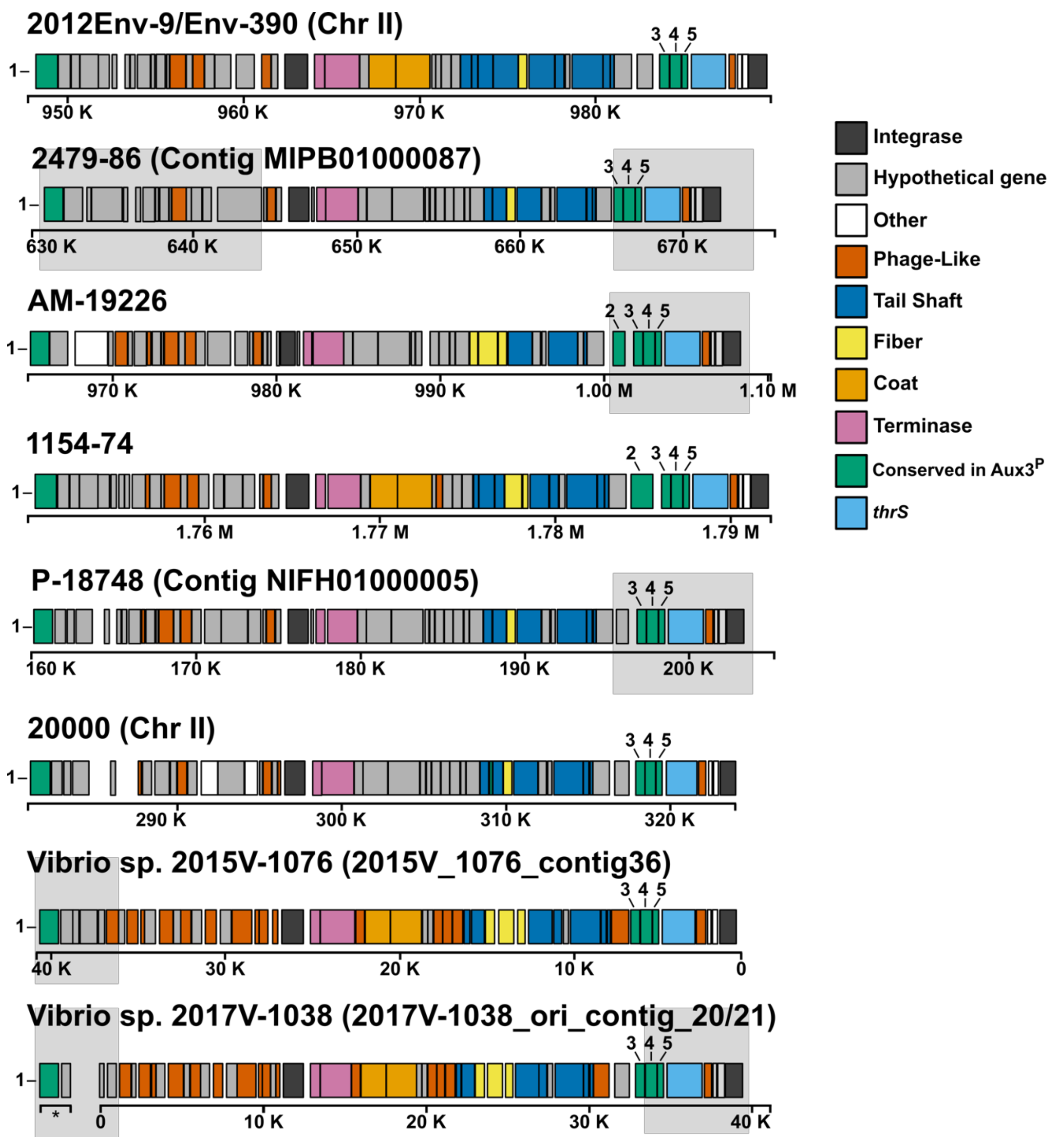

Supplementary Fig. 5: Aux3^{E} strains encode putatively intact prophage elements. PHASTER genome diagrams showing predicted prophage regions from *int* (VCA0281) through the Aux3-extrinsic superintegron integrase *intI4* (VCA0291). Coding regions are coloured according to homology to broad categories of known phage genes. Genes conserved in the pandemic Aux3 module are indicated (1 = *int*, 2 = VCA0283, 3 = *PAAR2*, 4 = *tseH*, 5 = *tsiH*). Light grey boxes indicate regions not called by PHASTER but confirmed by sequence analysis. * indicates coding regions found on a separate contig. Strains are shown in order from most closely related to the pandemic clade to least closely related (Fig. 3).

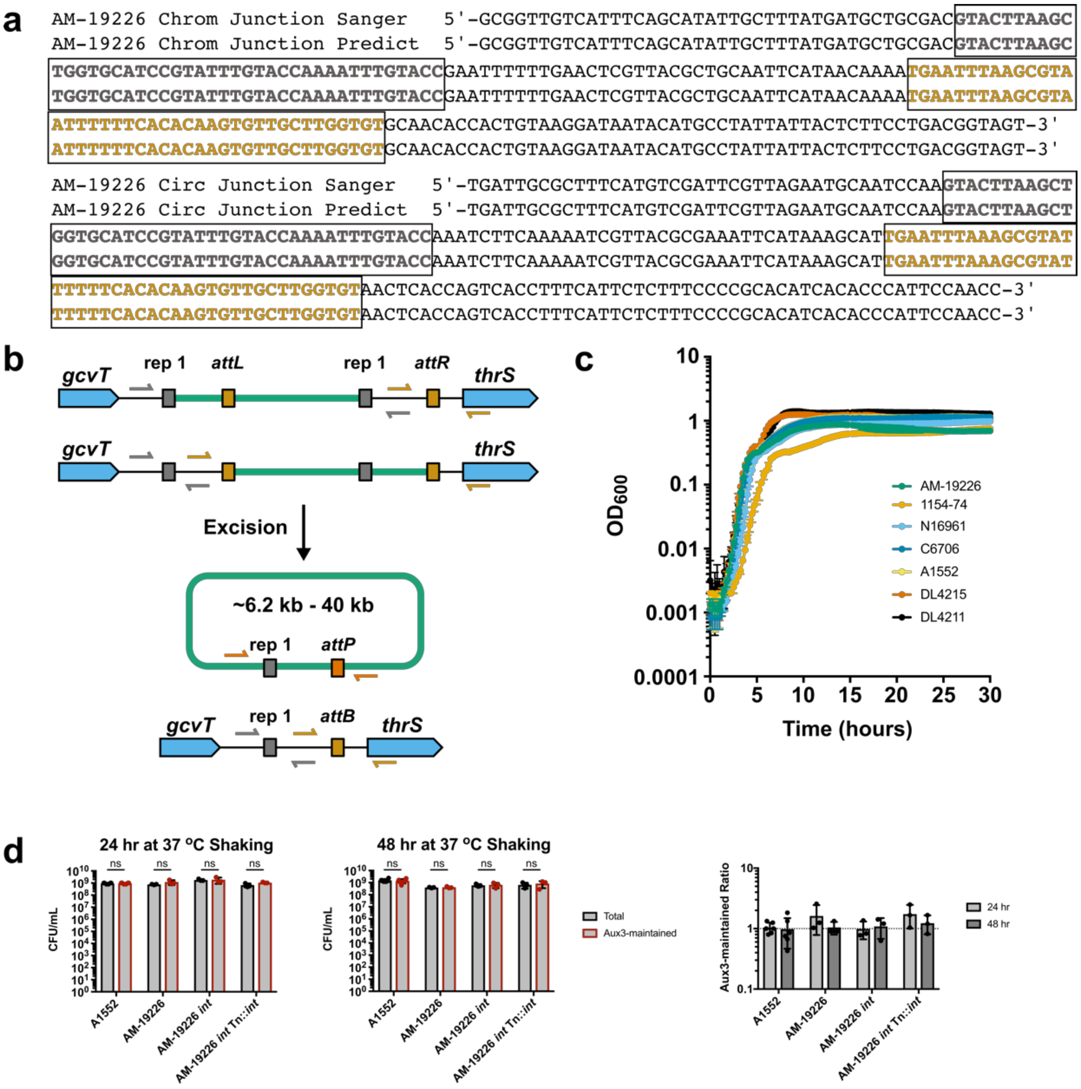

Supplementary Fig. 6: Excision PCR and qPCR controls and schematics. **a** MUSCLE alignment of predicted chromosomal and circular junction post Aux3 excision with Sanger sequence of inverted PCR bands from AM-19226. **b** Excision qPCR schematic. Grey arrows represent naïve repeat 1 primers. Orange arrows represent *attB_Aux3* primers. Dark orange arrows represent *attP_Aux3* primers. Primer binding sites for the two possible states for Aux3 integration (Top) and the excised state (Bottom) are shown. **c** Growth curves measured from three distinct experiments (n=3) for all strains used in PCR/qPCR experiments show that all strains grow approximately the same under the experimental conditions. Points represent the mean and error bars indicate ± SD. **d** Aux3 excision tracking experiments looking at Aux3-maintenance in long-term culture. Total CFU/mL (Rif^R for A1552 and Sm^R for AM-19226) and Aux3-maintained CFU/mL (Kan^R) are shown at 24 hr (left) and 48 hr (middle) timepoints. Results are from three distinct experiments (n=3). Horizontal bars represent the mean and error bars indicate ± SD. No significant difference is seen between Total and Aux3-maintained by 2way ANOVA with Sidak's multiple comparisons test for any of the tested strains (ns = not significant). Ratio of Aux3-

maintained CFU mL^{-1} to Total CFU mL^{-1} is also shown (right). **c-d** Source data are provided as a Source Data file.

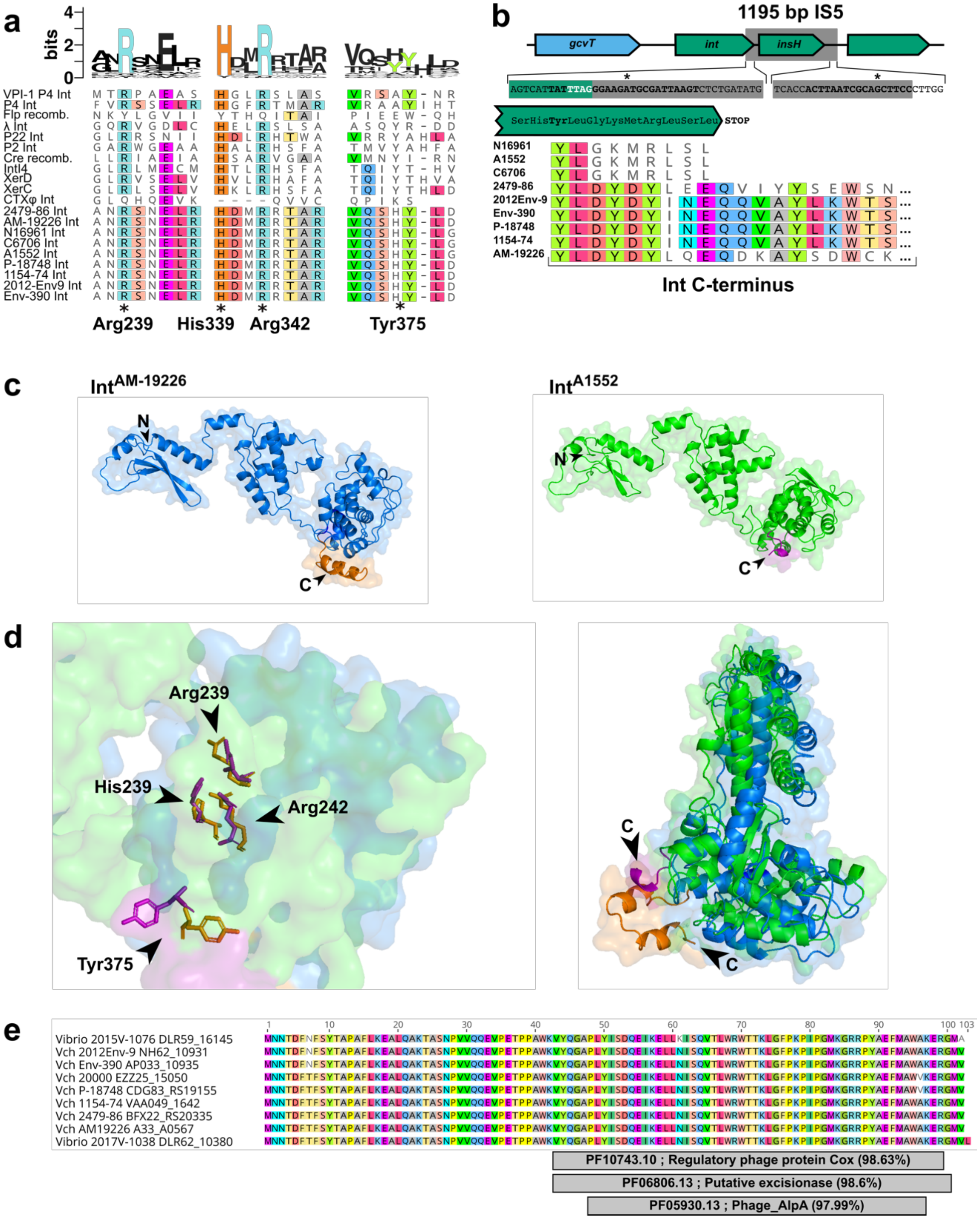

Supplementary Fig. 7: Reduced excision in Aux3^{P} is due to integrase truncation and loss of an RDF. **a** MUSCLE alignment of Aux3^{P} and Aux3^{E} integrases with known tyrosine recombinases. Three regions containing the four main catalytic residues[1] are shown with the known catalytic residues highlighted in the SeqLogo (top), by *, and by name below. **b** Schematic of IS5 element insertion into and blunting of the Aux3 integrase. The IS5 element is highlighted by a grey rectangle in the gene diagram (top). The black bolded nucleotide sequence indicates the canonical inverted repeats and a * marks the known disagreement in these repeats. The white bolded text indicates the IS5 target site (Pyr-

TA-Pur)[2] immediately after the catalytic Tyr (Y) residue. MUSCLE alignment (bottom) of the C-terminal amino acid sequence of $Aux3^{P}$ and $Aux3^{E}$ integrases indicates the introduction of a nonsense tail and premature STOP by the 5' sequence of the IS5 in pandemic strains. **c** Phyre2[3] intensive model of $Int^{AM-19226}$ (left) and Int^{A1552} (right) visualized with PyMol (v1.2r3pre). C-terminal tails after the catalytic tyrosine are coloured orange or magenta, respectively. N- and C-termini are highlighted by black arrows. **d** Overlay of surface models of $Int^{AM-19226}$ and Int^{A1552}. Catalytic residues are orange ($Int^{AM-19226}$) or magenta (Int^{A1552}) and highlighted by black arrows (left). Disparity in the C-terminal tail (right) is shown by a ribbon model. C-termini are highlighted by black arrows. **e** MUSCLE alignment of the amino acid sequence of the putative $Aux3^{E}$ RDF from each $Aux3^{E}$ strain. HHpred predicted domains are shown in grey boxes with the associated probability in parentheses.

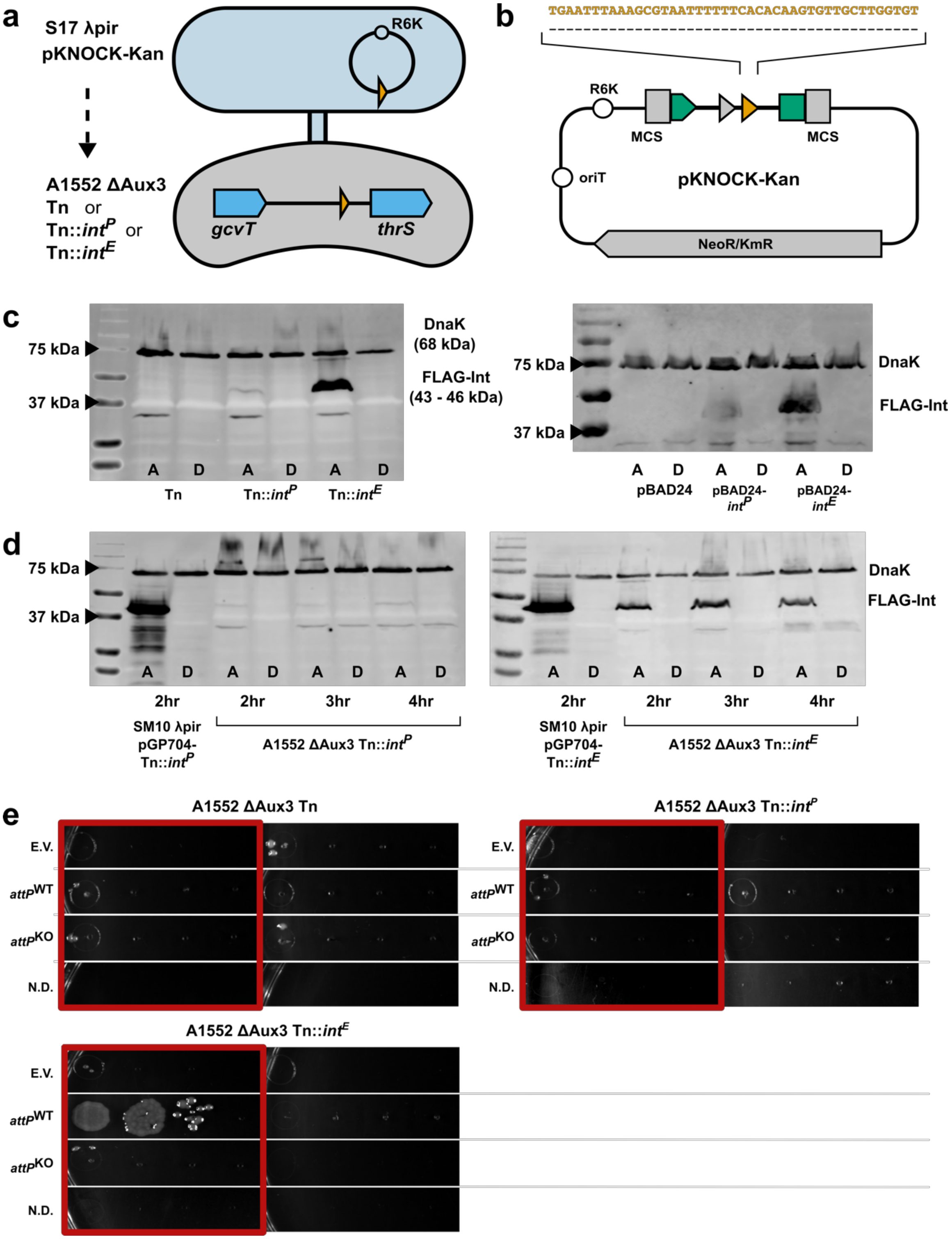

Supplementary Fig. 8: Arabinose induction of *FLAG-int*^{E} drives integration of pKNOCK-*attP*^{WT}. **a** Schematic of Aux3 transfer experiments showing conjugation between donor strain S17 λpir carrying variable pKNOCK-Kan vectors and recipient strain *V. cholerae* ΔAux3 with variable *int* genes expressed off of the mini Tn7 transposon. **b** Schematic of pKNOCK-Kan vectors carrying either WT *attP* or *attP* knockout fragments. **c** Western blot

analysis of 4 hr integrase induction in *V. cholerae* A1552 ΔAux3 strains from the mini Tn7 transposon (left) and A1552 strains from a multi-copy pBAD24 vector (right). A = 0.1% arabinose. D = 0.1% dextrose. **d** Western blot analysis of integrase induction in *V. cholerae* ΔAux3 strains and their respective SM10 λpir parental strains over a time course from 2 to 4 hours. **e** Representative images from cointegrate dilution plates from Aux3 transfer experiments (Fig. 5). Images highlighted in red are from arabinose-induced experiments. E.V. = S17 λpir;pKNOCK-Kan; *attP*^{WT} = S17 λpir;pKNOCK-*attP*^{WT}; *attP*^{KO} = S17 λpir;pKNOCK-*attP*^{KO}; N.D. = No Donor. **c-d** Blot images are representative of at least three distinct experiments (n=3). **c-e** Source data are provided as a Source Data file.

Supplementary Table 1. Bacterial Strains and Plasmids

Strain or Plasmid	Genotype*/Description	Internal Strain Ref.	Reference
DH5α λpir	F$^-$ endA1 glnV44 thi-1 recA1 relA1 gyrA96 deoR nupG φ80lacZΔM15 Δ(lacZYA-argF) U169 hsdR17 (r_K^- m_K^+) phoA, λ$^-$	FJS010	[4]
SM10 λpir	thi-1 thr leu tonA lacY supE recA::RP4-2-Tc::Mu, Kmr (λpir); Kan^R	FJS011	[5]
S17 λpir	Tpr Smr recA thi pro hsdR2M1 RP4:2-Tc:Mu:Kmr Tn7 (λpir); Sm^R	FJS275	[5]
SAD033	*V. cholerae* strain carrying spectinomycin resistance cassette for MuGENT cloning	FJS028	(Ankur Dalia)
N16961	WT O1 El Tor Pandemic Strain; Aux3^P; Sm^R	FJS002	[6]
C6706	WT O1 El Tor Pandemic Strain; Aux3^P; Sm^R	FJS005	[7]
A1552	WT O1 El Tor Pandemic Strain; Aux3^P; Rif^R	FJS024	[8]
AM-19226 Δ*endo*	O39 Clinical Isolate deleted for the type II restriction endonuclease TdeIII; Aux3^E; Sm^R	FJS021	[9]
1154-74	WT O49 Environmental Isolate; Aux3^E; Sm^R	FJS038	[10]
DL4215	WT Environmental Isolate; Aux3-naïve; Sm^R	FJS031	[11]
DL4211	WT Environmental Isolate; Aux3-naïve; Rif^R	FJS027	[11]
A1552 Δ*int*::Kan	A1552 deleted for VCA0281; Rif^R, Kan^R	FJS189	This study
A1552 ΔIS5	A1552 deleted of the 1195 bp IS5^{Aux3} element including VCA0282; Rif^R	FJS138	This study
A1552 Δ*int*-*insH*::Kan	A1552 deleted for VCA0281-VCA0282; Rif^R, Kan^R	FJS182	This study
A1552 Δ*int*::Kan Tn	A1552 Δ*int*::Kan containing empty mini-Tn7; Rif^R, Kan^R, Gent^R	FJS209	This study
A1552 Δ*int*::Kan Tn::*int*^{A1552}	A1552 Δ*int*::Kan containing mini-Tn7-P$_{end}$-*int*^{A1552}; Rif^R, Kan^R, Gent^R	FJS210	This study
A1552 Δ*int*::Kan Tn::*int*$^{AM-19226}$	A1552 Δ*int*::Kan containing mini-Tn7-P$_{end}$-*int*$^{AM-19226}$; Rif^R, Kan^R, Gent^R	FJS211	This study
A1552 Δ*int*::Kan Tn::*int*$^{1154-74}$	A1552 Δ*int*::Kan containing mini-Tn7-P$_{end}$-*int*$^{1154-74}$; Rif^R, Kan^R, Gent^R	FJS212	This study
AM-19226 Δ*endo* Δ*int*::Kan	AM-19226 Δ*endo* deleted for VCA0281 equivalent; Sm^R, Kan^R	FJS206	This study
AM-19226 Δ*endo* Δ*int*::Kan Tn	AM-19226 Δ*int*::Kan containing empty mini-Tn7; Sm^R, Kan^R, Gent^R	FJS207	This study
AM-19226 Δ*endo* Δ*int*::Kan Tn::*int*$^{AM-19226}$	AM-19226 Δ*int*::Kan containing mini-Tn7-P$_{end}$-*int*$^{AM-19226}$; Sm^R, Kan^R, Gent^R	FJS208	This study
AM-19226 Δ*endo* Δ*int*::Kan Tn::*int*^{A1552}	AM-19226 Δ*int*::Kan containing mini-Tn7-P$_{end}$-*int*^{A1552}; Sm^R, Kan^R, Gent^R	FJS229	This study
A1552;pBAD24	A1552 carrying empty pBAD24	FJS099	This study
A1552;pBAD24-*xis*	A1552 carrying pBAD24-*xis*	FJS381	This study

A1552 Δ*int*::Kan Tn; pBAD24	A1552 Δ*int*::Kan Tn carrying empty pBAD24	FJS375	This study
A1552 Δ*int*::Kan Tn; pBAD24-*xis*	A1552 Δ*int*::Kan Tn carrying pBAD24-*xis*	FJS376	This study
A1552 Δ*int*::Kan Tn::*int*^{A1552}; pBAD24	A1552 Δ*int*::Kan Tn::*int*^{A1552} carrying empty pBAD24	FJS377	This study
A1552 Δ*int*::Kan Tn::*int*^{A1552}; pBAD24-*xis*	A1552 Δ*int*::Kan Tn::*int*^{A1552} carrying pBAD24-*xis*	FJS378	This study
A1552 Δ*int*::Kan Tn::*int*$^{AM-19226}$; pBAD24	A1552 Δ*int*::Kan Tn::*int*$^{AM-19226}$ carrying empty pBAD24	FJS379	This study
A1552 Δ*int*::Kan Tn::*int*$^{AM-19226}$; pBAD24-*xis*	A1552 Δ*int*::Kan Tn::*int*$^{AM-19226}$ carrying pBAD24-*xis*	FJS380	This study
S17 λpir; pKNOCK-*attP*^{WT}	Conjugative *E. coli* carrying pKNOCK-*attP*^{WT}	FJS276	This study
S17 λpir; pKNOCK-*attP*^{KO}	Conjugative *E. coli* carrying pKNOCK-*attP*^{KO}	FJS277	This study
S17 λpir; pKNOCK-Kan	Conjugative *E. coli* carrying empty pKNOCK-Kan	FJS278	This study
A1552 ΔAux3	A1552 deleted for Aux3 by homologous recombination with the DL4211 naïve *attB* region (MuGENT); Rif^{R}	FJS139	This study
A1552 ΔAux3 Tn	A1552 ΔAux3 containing empty mini-Tn7; Rif^{R}, Gent^{R}	FJS213	This study
A1552 ΔAux3 Tn::*int*^{P}	A1552 ΔAux3 containing mini-Tn7-*araC*-P_{BAD}-FLAG-*int*^{A1552}; Rif^{R}, Gent^{R}	FJS266	This study
A1552 ΔAux3 Tn::*int*^{E}	A1552 ΔAux3 containing mini-Tn7-*araC*-P_{BAD}-FLAG-*int*$^{AM-19226}$; Rif^{R}, Gent^{R}	FJS267	This study
A1552; pBAD24-*int*^{P}	A1552 carrying pBAD24-FLAG-*int*^{A1552}; Rif^{R}, Amp^{R}	FJS340	This study
A1552; pBAD24-*int*^{E}	A1552 carrying pBAD24-FLAG-*int*$^{AM-19226}$; Rif^{R}, Amp^{R}	FJS341	This study
Plasmids			
pUC19-*lacZ*::Spec^{R}	pUC19 vector carrying spectinomycin resistance cassette inserted in between 3kb flanks homologous to *V. cholerae lacZ* and the surrounding sequence; Amp^{R}, Spec^{R}	FJS068	This study
pUC19-ΔIS5	pUC19 vector carrying 3kb upstream and downstream flanks of the Aux3 IS5 element for IS5 deletion; Amp^{R}	FJS085	This study
pCVD442-*lacZ*^{WT}	oriR6K, pCVD442 *sacB* counter-selectable suicide cloning vector carrying a WT copy of *V. cholerae lacZ* for curing of spectinomycin resistance cassette	FJS106	This study
pKD13	pKD vector for the amplification of FRT-flanked kanamycin cassette; Amp^{R}, Kan^{R}	FJS159	[12]
pCVD442-Δ*int*::Kan^{FRT}	oriR6K, pCVD442 *sacB* counter-selectable suicide cloning vector carrying an FRT-Kan-FRT flanked by 1000 bp homology arms surrounding VCA0281	FJS160	This study

pCVD442-Δ*int*-*insH*::Kan^{FRT}	oriR6K, pCVD442 *sacB* counter-selectable suicide cloning vector carrying an FRT-Kan-FRT flanked by 1000 bp homology arms surrounding VCA0281-VCA0282	FJS162	This study
pCVD442-Δ*int*$^{AM\text{-}19226}$::Kan^{FRT}	oriR6K, pCVD442 *sacB* counter-selectable suicide cloning vector carrying an FRT-Kan-FRT flanked by 1000 bp homology arms surrounding the AM-19226 VCA0281 homolog	FJS188	This study
pUX-BF-13	oriR6K, helper plasmid with Tn7 transposase; Amp^R	FJS032	[13]
pGP704-mTn7-minus SacI	pGP704 with mini-Tn7 insertion; Amp^R, Gent^R	FJS074	[14]
pGP704-mTn7-*int*^{A1552}	pGP704 with mini-Tn7 carrying P_{end}-driven *int*^{A1552}; Amp^R, Gent^R	FJS173	This study
pGP704-mTn7-*int*$^{AM\text{-}19226}$	pGP704 with mini-Tn7 carrying P_{end}-driven *int*$^{AM\text{-}19226}$; Amp^R, Gent^R	FJS171	This study
pGP704-mTn7-*int*$^{1154\text{-}74}$	pGP704 with mini-Tn7 carrying P_{end}-driven *int*$^{1154\text{-}74}$; Amp^R, Gent^R	FJS172	This study
pBAD24-*xis*	pBAD24 expression vector carrying a copy of *xis* from AM-19226	FJS370	This study
pGP704-mTn7-*araC*-P_{BAD}-FLAG-*int*^{A1552}	pGP704 with mini-Tn7 carrying *araC* and P_{BAD}-driven *int*^{A1552} with an N-terminal FLAG tag; Amp^R, Gent^R	FJS260	This study
pGP704-mTn7-*araC*-P_{BAD}-FLAG-*int*$^{AM\text{-}19226}$	pGP704 with mini-Tn7 carrying *araC* and P_{BAD}-driven *int*$^{AM\text{-}19226}$ with an N-terminal FLAG tag; Amp^R, Gent^R	FJS261	This study
pBAD24-FLAG-*int*^{A1552}	pBAD24 expression vector carrying an N-terminal FLAG-tagged copy of *int*^{A1552}	FJS333	This study
pBAD24-FLAG-*int*$^{AM\text{-}19226}$	pBAD24 expression vector carrying an N-terminal FLAG-tagged copy of *int*$^{AM\text{-}19226}$	FJS334	This study
pUC19-*attP*	pUC19 vector with inserted 727 bp region including Aux3 *attP*	FJS216	This study
pKNOCK-Kan	oriR6K, knock-in plasmid; Kan^R	FJS269	[15]
pKNOCK-*attP*^{WT}	pKNOCK-Kan with inserted 727 bp region including Aux3 *attP*	FJS273	This study
pKNOCK-*attP*^{KO}	pKNOCK-Kan with inserted 684 bp region with deletion of Aux3 *attP*	FJS274	This study

*VC numbers as reported in [6]

Supplementary Table 2. Primers

Name	Sequence
P1	GCGGTTGTCATTTCAGCATATT
P2	CTGTGGCTTATCTCAGTCTTACC
P2.2	GAAAGGTGACTGGTGAGTTACA
P3	CAGCAACGGGTCTCAGTATT
P3.2	GTGCTCTTGGCTACGTTTCT
P4	TGACTACCGTCAGGAAGAGTAA
Pnaive_rep_1-F	GCGGTTGTCATTTCAGCATATT
Pnaive_rep_1-R	GAATTGCAGCGTAACGAGTTC
PattB_Aux3-F	CTCGTTACGCTGCAATTCATAAC
PattB_Aux3-R	TGACTACCGTCAGGAAGAGTAA
PattP_Aux3-F	GATTGCGCTTTCATGTCGAT
PattP_Aux3-R	GGAAAGAGAATGAAAGGTGACTG
MuGENT	
lacZ-Upstream_pUC19-SmaI_F	tgaattcgagctcggtacccACCCTAAGCGGTTCAATTTTG
lacZ-Upstream_Spec_R	ggatccccggaatTAACGATGTGCGGGTTTTG
Spec_*lacZ*-Upstream_F	ccgcacatcgttaATTCCGGGGATCCGTCGAC
Spec_*lacZ*-Downstream_R	cggcagtgccattTGTAGGCTGGAGCTGCTTC
lacZ-Downstream_Spec_F	cagctccagcctacaAATGGCACTGCCGTACAC
lacZ-Downstream_pUC19-SmaI_R	gtcgactctagaggatccccTGATCCGATGATCTTTTCG
ABD334	AGTGCTCCGGACTCTTTGCTCTG
ABD335	CACTGCTCACTAGCGATGCAGTG
IS5-Upstream_pUC19-SmaI_F	gtcgactctagaggatccccgggGATCCTTTGGGTCACCGC
IS5-Upstream_R	attttccaagCTAAATAATGACTCTGAACACCAGTATG
IS5-Downstream_F	cattatttagCTTGGAAAATTGCGGGAG
IS5-Downstream_pUC19-SmaI_R	tgaattcgagctcggtacccgggAAACTGTTCAATATACGCGC
IS5-KO_Verification_F	CCAACCGCAGTAACGAATTG
IS5-KO_Verification_R	CCAGATCCTAATGTACCGTCTC
Aux3_KO_6kb_F	GCAGAGCAAGCATCACAG
Aux3_KO_6kb_R	ACCAGCTTTAATAACTGCTTG
lacZ-WT-6kb_pCVD442-SmaI_F	atgcgatatcgagctctcccgggACCCTAAGCGGTTCAATTTTG
lacZ-WT-6kb_pCVD442-SmaI_R	taacaatttgtggaattcccgggTGATCCGATGATCTTTTCG
Allelic Exchange	
int-Upstream_pCVD442-SmaI_F	accgcatgcgatatcgagctctcccgggCCTGCTGATAAAGCGGCG
int-Upstream_KanFRT_R	tccagcctacCTTGCATCAGAAAATTTGGTACAAATTTG
KanFRT_*int*-Upstream_F	ctgatgcaagGTAGGCTGGAGCTGCTTC
KanFRT_*int*-Downstream_R	tgatctcataATTCCGGGGATCCGTCGAC
int-Downstream_KanFRT_F	tccccggaatTATGAGATCATAGCAACCATC
int-Downstream_pCVD442-SmaI_R	gcggataacaatttgtggaattcccgggCGTCGTATCAGTTGGTCG
int-KO-Verification_F	AACTCGTTACGCCGCTATTT
int-KO-Verification_R	GTCACCTCATCCTTGCTGTT
KanFRT_*insH*-Downstream_R	ttggcggattATTCCGGGGATCCGTCGAC
insH-Downstream _KanFRT_F	tccccggaatAATCCGCCAATAGCCGGAG
insH-Downstream_pCVD442-SmaI_R	gcggataacaatttgtggaattcccgggTTGATGCTCTGTCCATCC
int-insH-KO-Verification_F	AACTCGTTACGCCGCTATTT
int-insH-KO-Verification_R	CCAGATCCTAATGTACCGTCTC
int$^{AM-19226}$-Upstream_pCVD442-SmaI_F	accgcatgcgatatcgagctctcccgggAGTGCCTGCTGATAAAGC

int$^{AM-19226}$-Upstream _KanFRT_R	gctccagcctacACTCACCTTGCATCAGAAAATTTG
KanFRT_*int*$^{AM-19226}$-Upstream _F	atgcaaggtgagtGTAGGCTGGAGCTGCTTC
KanFRT_*int*$^{AM-19226}$-Downstream _R	tatgtttatccatATTCCGGGGATCCGTCGAC
int$^{AM-19226}$-Downstream_KanFRT_F	gatccccggaatATGGATAAACATAGTAATAGTGAC
int$^{AM-19226}$-Downstream_pCVD442-SmaI_R	gcggataacaatttgtggaattcccgggTTAAATTTGAACAGTCAAAA CTATAG
int$^{AM-19226}$-KO_Verification_F	TGTAACTCACCAGTCACCTTTC
int$^{AM-19226}$-KO_Verification_R	GACAACTCATTCCAGCCATAGA
Transcomplementation	
All-*int*-P$_{end}$_pGP704-mTn7-NotI_F	ggatccacgcgtcttaaggcTTTCTTCAGTGTCATTCTTATG
int^{A1552}_pGP704-mTn7-NotI_R	cccgacgggcccggtaccgcTCAGAGACTTAATCGCATC
int$^{AM-19226}$_pGP704-mTn7-NotI_R	cccgacgggcccggtaccgcTTATCCATTATAATAAATTCCTAGAATAC
int$^{1154-74}$_pGP704-mTn7-NotI_R	cccgacgggcccggtaccgcTTAGAAAATAATACTTGTCCACTTC
FLAG-All-*int*_pGP704-mTn7-araC-P$_{BAD}$-NcoI_F	ggctagcaggaggaattcacc***atggactacaaagacgatgacgacaag*** GCAATAACGGATGCATGG
int^{A1552}_pGP704-mTn7-araC-P$_{BAD}$-NcoI_R	tctagaggatccccgggtacTCAGAGACTTAATCGCATCTTC
int$^{AM-19226}$_pGP704-mTn7-araC-P$_{BAD}$-NcoI_R	tctagaggatccccgggtacTTATCCATTATAATAAATTCCTAGAATACTC
*vefD*_pBAD24-SmaI_F	aggaggaattcaccatggtaATGAACAATACCGATTTTACTTTC
*vefD*_pBAD24-SmaI_R	caggtcgactctagaggatcTCATACCATCCCGCGTTC
Donor Vectors	
Aux3_Frag1_F	aagcgtatttTTTCACACAAGTGTTGCTTG
Aux3_Frag1_*attP*_KO_F	cataaagcatAACTCACCAGTCACCTTTCATT
Aux3_Frag1_R	TGCCGATGAAGAAGATGTATG
Aux3_Frag2_F	AACAAGACAAGTCTGAACG
Aux3_Frag2_R	ttgtgtgaaaAAATACGCTTTAAATTCAATG
Aux3_Frag2_*attP*_KO_R	ctggtgagttATGCTTTATGAATTTTGCG
*attP*_pKNOCK-Kan-SmaI_F	gctctagaactagtggatccCAGCAACGGGTCTCAGTATTG
*attP*_pKNOCK-Kan-SmaI_R	ttgatatcgaattcctgcagCTGTGGCTTATCTCAGTCTTAC
Other	
pUC19_Insert_F	GGAAACAGCTATGACCATGATTAC
pUC19_Insert_R	GGGTAACGCCAGGGTTT
pCVD442_insert_F	ACTAAATAATAGTGAACGGCAGGTA
pCVD442_insert_R	GTGAGCGGATAACAATTTGTGG
pBAD24_insert_F	GGCGTCACACTTTGCTATG
pBAD24_insert_R	GTTCTGATTTAATCTGTATCAGGCT
pKNOCK_ins_F	GGGATGTAACGCACTGAGAA
pKNOCK_ins_R	CGGATTCACCACTCCAAGAA
pUC19_*attP*_gib_F	gtcgactctagaggatccccgggCAGCAACGGGTCTCAGTATTG
pUC19_*attP*_gib_R	tgaattcgagctcggtacccgggCTGTGGCTTATCTCAGTCTTAC

* Gibson overlaps are shown as lowercase letters. When Gibson assembly involves three fragments, primers are named such that the first half of the name describes the amplified region and the second half describes the region to which the overlap is complementary.

Supplementary Table 3. Genomes Used In This Study

Organism	Strain	Serotype	Biotype	Ref Seq	Assembly Level
V. cholerae	N16961	O1	El Tor	GCF_000006745.1	Complete
V. cholerae	A1552	O1	El Tor	GCF_002892855.1	Complete
V. cholerae	M66-2	O1	El Tor	GCF_000021605.1	Complete
V. cholerae	MAK 757	O1	El Tor	GCF_000153865.1	Scaffold
V. cholerae	C6706	O1	El Tor	GCF_000237785.1	Contig
V. cholerae	CIRS101	O1	El Tor	GCF_000175695.1	Contig
V. cholerae	CP1041	O1	El Tor	GCF_000279245.1	Contig
V. cholerae	MJ-1236	O1	El Tor	GCF_000022585.1	Complete
V. cholerae	2010EL-1786	O1	El Tor	GCF_000166455.1	Complete
V. cholerae	HC-07A1	O1	El Tor	GCF_000318485.2	Contig
V. cholerae	BX 330286	O1	El Tor	GCF_000174335.1	Contig
V. cholerae	O395	O1	Classical	GCF_000016245.1	Complete
V. cholerae	V52	O37		GCF_000167935.2	Scaffold
V. cholerae	MS6	O1		CF_000829215.1	Complete
V. cholerae	AM-19226	O39		GCF_000153785.2	Scaffold
V. cholerae	PA1849	O1	Classical	NA	
V. cholerae	CA401	O1	Classical	NA	
V. cholerae	A76	O1	Classical	GCF_001259495.1	Scaffold
V. cholerae	A60	O1	Classical	GCF_001248195.1	Scaffold
V. cholerae	A68	O1	Classical	GCF_001259635.1	Scaffold
V. cholerae	A111	O1	Classical	GCA_001253495.1 (GenBank)	Scaffold
V. cholerae	A59	O1	Classical	GCF_001254535.1	Scaffold
V. cholerae	A49	O1	Classical	GCA_001253835.1 (GenBank)	Scaffold
V. cholerae	A57	O1	Classical	GCA_001250255.1 (GenBank)	Scaffold
V. cholerae	A51	O1	Classical	GCA_001253435.1 (GenBank)	Scaffold
V. cholerae	A46	O1	Classical	GCF_001259555.1	Scaffold
V. cholerae	NIH41	O1	Classical	GCF_000736865.1	Contig
V. cholerae	RC27	O1	Classical	GCF_000176395.1	Contig
V. cholerae	A279	O1	Classical	GCA_001253555.1 (GenBank)	Scaffold
V. cholerae	A61	O1	Classical	GCF_001250935.1	Scaffold
V. cholerae	A50	O1	Classical	GCA_001254735.1 (GenBank)	Scaffold
V. cholerae	A103	O1	Classical	GCA_001254575.1 (GenBank)	Scaffold
V. cholerae	A70	O1	Classical	GCF_001248905.1	Scaffold
V. cholerae	GP16	O1	Classical	GCF_001251495.1	Scaffold
V. cholerae	GP8	O1	Classical	GCF_001253575.1	Scaffold
V. cholerae	A389	O1	Classical	GCA_001259795.1 (GenBank)	Scaffold
V. cholerae	A66	O1	Classical	GCF_001260915.1	Scaffold
V. cholerae	2740-80	O1		GCF_000168915.2	Scaffold
V. cholerae	NCTC8457	O1	El Tor	GCF_000153945.1	Scaffold
V. cholerae	HC38-A1	O1	El Tor	GCF_000221485.1	Scaffold
V. cholerae	HC33-A2	O1	El Tor	GCF_000234885.1	Scaffold
V. cholerae	HC32-A1	O1	El Tor	GCF_000234905.1	Scaffold
V. cholerae	FC1225	O139		GCF_002194335.1	Contig

V. cholerae	FC2273	O139		GCF_002194215.1	Contig
V. cholerae	FC2271	O139		GCF_002194235.1	Contig
V. cholerae	FC1341	O139		GCF_002194265.1	Contig
V. cholerae	FC3611a	O139		GCF_002194165.1	Contig
V. cholerae	FC1384	O139		GCF_002194245.1	Contig
V. cholerae	FC3611b	O139		GCF_002194185.1	Contig
V. cholerae	FC1105	O139		GCF_002194295.1	Contig
V. cholerae	CP1041	O1	El Tor	GCF_000279245.1	Contig
V. cholerae	MO10	O139		GCF_000152425.1	Scaffold
V. cholerae	FC1877	O139		GCF_002194155.1	Contig
V. cholerae	FC1817	O139		GCF_002194305.1	Contig
V. cholerae	IEC224	O1	El Tor	GCF_000250855.1	Complete
V. cholerae	A6	O1	El Tor	GCF_001255575.1	Scaffold
V. cholerae	1154-74	O49		GCF_000969235.1	Complete
V. cholerae	2479-86	O1		GCF_001857305.1	Contig
V. cholerae	2012Env-9	O1		GCF_000788715.2	Complete
V. cholerae	Env-390	O1		GCF_001854425.1	Complete
V. cholerae	20000	nonO1/O139		GCF_004328575.1	Complete
V. cholerae	P-18748	nonO1/O139		GCF_002196055.1	Contig
V. cholerae	HE-39	nonO1/O139		GCF_000220765.2	Contig
V. cholerae	HC-43B1	O1		GCF_000279435.1	Contig
V. cholerae	TM11079-80	O1		GCF_000174255.1	Contig
V. cholerae	HE-45	nonO1/O139		GCF_000279285.1	Contig
V. cholerae	1587	O12		GCF_000168895.2	Scaffold
V. cholerae	12129(1)	O1		GCF_000174115.1	Contig
V. cholerae	TMA21	nonO1/O139		GCF_000174295.1	Contig
V. cholerae	DL4211	O123		GCF_001953365.1	Scaffold
V. cholerae	623-39	nonO1/O139		GCF_000154005.2	Scaffold
V. cholerae	HE-25	nonO1/O139		GCF_000279265.1	Contig
V. cholerae	DL4215	O113		GCF_001953375.1	Scaffold
V. cholerae	MZO-2	O14		GCF_000153985.2	Scaffold
V. cholerae	MZO-3	O37		GCF_000168935.2	Scaffold
V. cholerae	571-88	O105		GCF_000736945.1	Contig
V. cholerae	234-93	O141		GCF_000737005.1	Contig
V. cholerae	3568-07	O141		GCF_001857505.1	Contig
V. cholerae	V51	O141		GCF_000152465.2	Scaffold
V. cholerae	CP1110	O75		GCF_000387585.1	Contig
V. cholerae	CP1111	O75		GCF_000387625.1	Contig
V. cholerae	CP1112	O75		GCF_000387645.1	Contig
V. cholerae	CP1113	O75		GCF_000387665.1	Contig
V. cholerae	CP1114	O75		GCF_000387685.1	Contig
V. cholerae	CP1115	O75		GCF_000387605.1	Contig
V. cholerae	CP1116	O75		GCF_000387725.1	Scaffold
V. cholerae	CP1117	O75		GCF_000387705.1	Contig
Vibrio sp.	2015V-1076			GCF_003311815.1	Contig
Vibrio sp.	2017V-1038			GCF_003311805.1	Contig
Vibrio sp.	2017V-1070			GCF_003311865.1	Contig
Vibrio sp.	2016V-1062			GCF_003311825.1	Contig
Vibrio sp.	2017V-1085			GCF_003311895.1	Contig
Vibrio sp.	2523-88			GCF_003311755.1	Contig
Vibrio sp.	2016V-1018			GCF_003312035.1	Contig
Vibrio sp.	2017V-1124			GCF_003311885.1	Contig
V. mimicus	SX-4			GCF_000222145.1	Scaffold

125

Supplementary Table 4. Aux3 Enrichment Analysis

	tseL/vasX/vgrG3 Grades ≥ 99%	*tseL/vasX/vgrG3* Grades < 99%	Total
tseH Grade ≥ 99%	461	1	462
tseH Grade < 99%	86	24	110
Total	547	25	572
Two-tailed Fisher's Exact Test: $p = 2.2 \times 10^{-16}$			

Supplementary Table 5. Aux3 Transfer Experiment Cointegrate Formation Frequencies

Experiment		Donor CFU/mL	Recipient CFU/mL	Cointegrate CFU/mL	Cointegrate Formation Frequency
E.V. x Tn	A	4.07E+08 ± 1.80E+08	1.03E+09 ± 3.00E+08	199.00 ± 0.00	2.08E-07 ± 7.32E-08
	D	2.73E+08 ± 6.43E+07	2.47E+09 ± 1.79E+09	199.00 ± 0.00	1.48E-07 ± 1.50E-07
E.V. x Tn::*int*^{P}	A	2.13E+08 ± 9.24E+07	1.00E+09 ± 3.46E+08	199.00 ± 0.00	2.21E-07 ± 9.57E-08
	D	2.25E+08 ± 1.36E+08	1.01E+09 ± 5.06E+08	199.00 ± 0.00	2.53E-07 ± 1.73E-07
E.V. x Tn::*int*^{E}	A	2.80E+08 ± 2.00E+07	3.32E+09 ± 4.07E+09	266.00 ± 116.05	2.22E-07 ± 1.74E-07
	D	3.47E+08 ± 1.21E+08	1.80E+09 ± 3.46E+08	199.33 ± 0.58	1.13E-07 ± 1.98E-08
attP^{WT} x Tn	A	2.07E+08 ± 4.16E+07	5.67E+08 ± 2.77E+08	266.00 ± 116.05	5.25E-07 ± 2.28E-07
	D	2.33E+08 ± 1.17E+08	1.53E+09 ± 1.03E+09	800.00 ± 346.41	1.05E-06 ± 1.26E-06
attP^{WT} x Tn::*int*^{P}	A	2.20E+08 ± 7.21E+07	5.13E+08 ± 1.03E+08	666.67 ± 115.47	1.33E-06 ± 2.83E-07
	D	2.47E+08 ± 1.03E+08	2.27E+09 ± 1.42E+09	199.67 ± 0.58	1.17E-07 ± 7.47E-08
attP^{WT} x Tn::*int*^{E}	A	1.40E+08 ± 7.21E+07	1.13E+09 ± 3.16E+08	1.33E+05 ± 5.77E+04	1.22E-04 ± 4.79E-05
	D	1.47E+08 ± 1.01E+08	1.53E+09 ± 9.45E+08	733.00 ± 757.54	1.89E-07 ± 5.17E-08
attP^{KO} x Tn	A	2.73E+08 ± 7.02E+07	8.00E+08 ± 4.00E+08	266.33 ± 115.76	3.60E-07 ± 1.26E-07
	D	2.40E+08 ± 1.59E+08	2.07E+09 ± 1.01E+09	199.33 ± 0.58	1.19E-07 ± 7.06E-08
attP^{KO} x Tn::*int*^{P}	A	2.47E+08 ± 5.77E+07	6.67E+08 ± 4.62E+08	399.67 ± 200.50	8.89E-07 ± 6.74E-07
	D	2.07E+08 ± 1.29E+08	2.67E+09 ± 1.90E+09	266.33 ± 115.76	1.37E-07 ± 9.66E-08
attP^{KO} x Tn::*int*^{E}	A	2.33E+08 ± 5.77E+07	8.67E+08 ± 5.03E+08	333.00 ± 231.23	4.64E-07 ± 3.06E-07
	D	1.57E+08 ± 1.45E+08	2.63E+09 ± 2.65E+09	199.33 ± 0.58	1.82E-07 ± 1.92E-07
Tn only	A	199.67 ± 0.58	7.67E+08 ± 3.79E+08	199.00 ± 0.00	2.99E-07 ± 1.20E-07
	D	999.67 ± 1216.88	1.53E+09 ± 7.02E+08	199.67 ± 0.58	2.05E-07 ± 1.70E-07
Tn::*int*^{P} only	A	266.00 ± 116.05	9.20E+08 ± 4.85E+08	199.00 ± 0.00	2.95E-07 ± 2.23E-07
	D	266.00 ± 116.05	2.20E+09 ± 1.25E+09	199.00 ± 0.00	1.11E-07 ± 5.53E-08
Tn::*int*^{E} only	A	199.00 ± 0.00	8.67E+08 ± 2.31E+08	199.00 ± 0.00	2.43E-07 ± 7.66E-08
	D	999.67 ± 1385.93	1.80E+09 ± 3.46E+08	199.00 ± 0.00	1.14E-07 ± 2.46E-08

Supplementary References

1. Esposito, D. & Scocca, J. The integrase family of tyrosine recombinases: evolution of a conserved active site domain. *Nucleic Acids Res.* **25**, 3605–3614 (1997).
2. Schoner, B. & Kahn, M. The nucleotide sequence of IS5 from Escherichia coli. *Gene* **14**, 165–174 (1981).
3. Kelley, L., Mezulis, S., Yates, C., Wass, M. & Sternberg, M. The Phyre2 web portal for protein modeling, prediction and analysis. *Nat. Protoc.* **10**, 845–858 (2015).
4. Platt, R., Drescher, C., Park, S.-K. & Phillips, G. Genetic System for Reversible Integration of DNA Constructs and lacZ Gene Fusions into the Escherichia coli Chromosome. *Plasmid* **43**, 12–23 (2000).
5. Simon, R., Priefer, U. & Pühler, A. A Broad Host Range Mobilization System for In Vivo Genetic Engineering: Transposon Mutagenesis in Gram Negative Bacteria. *Nat. Biotechnol.* **1**, 784–791 (1983).
6. Heidelberg, J. *et al.* DNA sequence of both chromosomes of the cholera pathogen Vibrio cholerae. *Nature* **406**, 477–483 (2000).
7. Thelin, K. & Taylor, R. Toxin-coregulated pilus, but not mannose-sensitive hemagglutinin, is required for colonization by Vibrio cholerae O1 El Tor biotype and O139 strains. *Infect. Immun.* **64**, 2853–2856 (1996).
8. Yildiz, F. & Schoolnik, G. Role of rpoS in stress survival and virulence of Vibrio cholerae. *J. Bacteriol* **180**, 773–784 (1998).
9. Tam, V., Serruto, D., Dziejman, M., Brieher, W. & Mekalanos, J. A Type III Secretion System in Vibrio cholerae Translocates a Formin/Spire Hybrid-like Actin Nucleator to Promote Intestinal Colonization. *Cell Host Microbe* **1**, 95–107 (2007).
10. Johnson, S. *et al.* Complete Genome Assemblies for Two Single-Chromosome Vibrio cholerae Isolates, Strains 1154-74 (Serogroup O49) and 10432-62 (Serogroup O27). *Genome Announc.* **3**, e00462-15 (2015).
11. Unterweger, D. *et al.* Constitutive Type VI Secretion System Expression Gives Vibrio cholerae Intra- and Interspecific Competitive Advantages. *Plos One* **7**, e48320 (2012).

12. Datsenko, K. & Wanner, B. One-step inactivation of chromosomal genes in Escherichia coli K-12 using PCR products. *Proc. Natl. Acad. Sci. U.S.A.* **97**, 6640–6645 (2000).
13. Bao, Y., Lies, D., Fu, H. & Roberts, G. An improved Tn7-based system for the single-copy insertion of cloned genes into chromosomes of gram-negative bacteria. *Gene* **109**, 167–168 (1991).
14. Müller, J., Miller, M., Nielsen, A., Schoolnik, G. & Spormann, A. vpsA- and luxO-independent biofilms of Vibrio cholerae. *Fems Microbiol. Lett.* **275**, 199–206 (2007).
15. Alexeyev, M. The pKNOCK series of broad-host-range mobilizable suicide vectors for gene knockout and targeted DNA insertion into the chromosome of gram-negative bacteria. *BioTechniques* **26**, 824–828 (1999).
