# Supplementary material for: Pandemic Vibrio cholerae shuts down site-specific recombination to retain an interbacterial defence mechanism
Source: Nat Commun. 2020 Dec 7;11:6246. doi: 10.1038/s41467-020-20012-7 (PMC7721734; doi:10.1038/s41467-020-20012-7)
Supplement: Supplementary file 3 — Description of Additional Supplementary Files [file 41467_2020_20012_MOESM3_ESM.pdf]

Description of Additional Supplementary Files

File Name: Supplementary Data 1

Description: Table of MegaBlast grades for *tseH*, *tseL*, *vasX*, *vgrG-3*, *tcpA*, and *ctxAB* in all analysed *V. cholerae* genomes.

File Name: Supplementary Data 2

Description: Functional predictions of coding regions in eight Aux3^{E} clusters using Prokka, NCBI CD-Search, and PHASTER.
