# Supplementary material for: Pandemic Vibrio cholerae shuts down site-specific recombination to retain an interbacterial defence mechanism
Source: Nat Commun. 2020 Dec 7;11:6246. doi: 10.1038/s41467-020-20012-7 (PMC7721734; doi:10.1038/s41467-020-20012-7)
Supplement: Supplementary file 6 — Reporting Summary [file 41467_2020_20012_MOESM6_ESM.pdf]

nature research

Corresponding author(s): Stefan Pukatzki

Last updated by author(s): Oct 26, 2020

Reporting Summary

Nature Research wishes to improve the reproducibility of the work that we publish. This form provides structure for consistency and transparency in reporting. For further information on Nature Research policies, see our Editorial Policies and the Editorial Policy Checklist.

Statistics

For all statistical analyses, confirm that the following items are present in the figure legend, table legend, main text, or Methods section.

n/a	Confirmed	
☐	☒	The exact sample size (n) for each experimental group/condition, given as a discrete number and unit of measurement
☐	☒	A statement on whether measurements were taken from distinct samples or whether the same sample was measured repeatedly
☐	☒	The statistical test(s) used AND whether they are one- or two-sided<br>*Only common tests should be described solely by name; describe more complex techniques in the Methods section.*
☒	☐	A description of all covariates tested
☐	☒	A description of any assumptions or corrections, such as tests of normality and adjustment for multiple comparisons
☐	☒	A full description of the statistical parameters including central tendency (e.g. means) or other basic estimates (e.g. regression coefficient) AND variation (e.g. standard deviation) or associated estimates of uncertainty (e.g. confidence intervals)
☐	☒	For null hypothesis testing, the test statistic (e.g. F, t, r) with confidence intervals, effect sizes, degrees of freedom and P value noted<br>*Give P values as exact values whenever suitable.*
☒	☐	For Bayesian analysis, information on the choice of priors and Markov chain Monte Carlo settings
☒	☐	For hierarchical and complex designs, identification of the appropriate level for tests and full reporting of outcomes
☒	☐	Estimates of effect sizes (e.g. Cohen's d, Pearson's r), indicating how they were calculated

Our web collection on statistics for biologists contains articles on many of the points above.

Software and code

Policy information about availability of computer code

Data collection	Quantitative PCR data (Fig. 4c,d) were collected in Bio-Rad CFX Manager 3.1.
Data analysis	Genomic analyses (sequence alignments, custom blast searches, etc.) were performed in Geneious Prime (v 2019.0.4). Sequence alignments were performed with either the Progressive Mauve Algorithm or MUSCLE (v3.8.425). Heatmaps were generated with R (v 3.3.2) using pam (R package cluster v2.1.0) and pheatmap (R package pheatmap v1.0.12). Phylogenetic trees were generated with Prokka (v1.12), Roary (v3.11.2), SNP-sites (v2.4.1), and RAxML (v 7.0.4) and visualized in TreeGraph 2 (v2.15.0-887 beta). Graphs and statistical analyses were generated using GraphPad Prism software (v 8.4.3). Putative protein structures were generated with Phyre2 web portal (www.sbg.bio.ic.ac.uk) and visualized with PyMol (v1.2r3pre). Proteins were predicted with NCBI Conserved Domain Search (https://www.ncbi.nlm.nih.gov/Structure/cdd/wrpsb.cgi) and HHPred (https://toolkit.tuebingen.mpg.de/tools/hhpred).

For manuscripts utilizing custom algorithms or software that are central to the research but not yet described in published literature, software must be made available to editors and reviewers. We strongly encourage code deposition in a community repository (e.g. GitHub). See the Nature Research guidelines for submitting code & software for further information.

Data

Policy information about availability of data

All manuscripts must include a data availability statement. This statement should provide the following information, where applicable:

- Accession codes, unique identifiers, or web links for publicly available datasets
- A list of figures that have associated raw data
- A description of any restrictions on data availability

The authors declare that all the data supporting the findings of this study are available within the paper and its supplementary information files. All genomes analysed in this study are publicly available from the PATRIC (https://www.patricbrc.org/) and NCBI RefSeq (https://www.ncbi.nlm.nih.gov/refseq/) databases.

April 2020

RefSeq accession numbers are provided in Supplementary Table 3. The source data underlying Figs. 4b-g, and 5a-d and Supplementary Figs. 6c-d, and 8c-e are provided as a Source Data file.

Field-specific reporting

Please select the one below that is the best fit for your research. If you are not sure, read the appropriate sections before making your selection.

- [x] Life sciences
- [ ] Behavioural & social sciences
- [ ] Ecological, evolutionary & environmental sciences

For a reference copy of the document with all sections, see nature.com/documents/nr-reporting-summary-flat.pdf

Life sciences study design

All studies must disclose on these points even when the disclosure is negative.

Sample size	No calculations were performed to determine sample size. Experiments were performed in biological triplicate based on previous experience with similar experiments and on previously published research using similar methods (Rajanna et al. J. Bacteriol. 2003, 10.1128/jb.185.23.6893-6901.2003; Murphy & Boyd et al. J. Bacteriol. 2008, 10.1128/JB.00562-07; Almagro-Moreno et al. BMC Microbiology, 10.1186/1471-2180-10-306; Carpenter et al. J. Bacteriol. 2016, 10.1128/JB.00704-15; Labbate et al. Sci. Rep. 2016, 10.1128/JB.00704-15;).
Data exclusions	No data were excluded from the manuscript.
Replication	All experiments were performed with independent replicates as described in the figure legends.
Randomization	Randomization is not applicable to this study as it uses bacterial strains.
Blinding	Blinding is not applicable to the majority of this study as it uses bacterial strains. When experiments required manual counts of colony forming units from different treatments (Fig. 5a,b,c and Supp Fig. 6d), CFUs were counted blind to strain and selective antibiotics to avoid bias in the counts.

Reporting for specific materials, systems and methods

We require information from authors about some types of materials, experimental systems and methods used in many studies. Here, indicate whether each material, system or method listed is relevant to your study. If you are not sure if a list item applies to your research, read the appropriate section before selecting a response.

Materials & experimental systems

n/a	Involved in the study
[x]	[ ] Antibodies
[x]	[ ] Eukaryotic cell lines
[x]	[ ] Palaeontology and archaeology
[x]	[ ] Animals and other organisms
[x]	[ ] Human research participants
[x]	[ ] Clinical data
[x]	[ ] Dual use research of concern

Methods

n/a	Involved in the study
[x]	[ ] ChIP-seq
[x]	[ ] Flow cytometry
[x]	[ ] MRI-based neuroimaging
